# Comparative study of dexmedetomidine versus fentanyl as adjuvants to bupivacaine in ultrasound-guided transversus abdominis plane block in patients undergoing radical cystectomy: a prospective randomised study

**DOI:** 10.1186/s12871-022-01877-1

**Published:** 2022-11-07

**Authors:** Dina Yehia Kassim, Hatem ElMoutaz Mahmoud, Dina Mahmoud Fakhry, Mariana AbdElSayed Mansour

**Affiliations:** Department of Anesthesiology, Surgical Intensive Care and Pain Management, Faculty of Medicine, Beni-Suef University, Beni-Suef, Egypt

**Keywords:** TAP block, Dexmedetomidine, Fentanyl, Bupivacaine, Radical cystectomy

## Abstract

**Background:**

Transversus abdominis plane (TAP) block is beneficial for pain management after conducting abdominal surgery.

**Objective:**

To compare the outcomes of dexmedetomidine and fentanyl, as adjuvants to bupivacaine, for ultrasound-guided TAP block analgesia among patients undergoing radical cystectomy for postoperative pain management.

**Methods:**

This prospective, randomised, comparative study included a total of 60 patients, who underwent radical cystectomy. Participants were randomly divided into three categories with 20 subjects each; group B had patients who received a single shot US-guided TAP block on each side with 20 ml of 0.25% bupivacaine + 2 ml normal saline; group BF had patients who received a single shot US-guided TAP block on each side with 20 ml of 0.25% bupivacaine + 1 µg/kg fentanyl dissolved in 2 ml normal saline and group BD had patients who received a single shot US-guided TAP block on each side with 20 ml of 0.25% bupivacaine + 1 µg/kg dexmedetomidine dissolved in 2 ml normal saline.The researchers recorded the time taken for first rescue analgesia, total analgesic dose in the first 24 h after surgery, patient satisfaction, sedation score, and postoperative complications.

**Results:**

The time taken for first rescue analgesia was significantly lengthier in group (BD) (8.90 ± 2.47) than (BF) (6.50 ± 1.43) and (B) (4.40 ± 1.05) groups. The total nalbuphine consumption, during the first 24 h, was significantly lower in (BD) (0.15 ± 0.00) group compared to (BF) (0.20 ± 0.07) and (B) (0.24 ± 0.08) groups.

**Conclusion:**

In comparison with fentanyl, as an adjuvant to bupivacaine, dexmedetomidine was found to be associated with prolonged postoperative analgesia, less postoperative pain scores and low opioid consumption.

**Trial registration:**

This study was registered at Clinical Trials.gov on 23 March 2020 (registration number: NCT04318158).

## Introduction

Radical cystectomy (RC) is the gold standard surgical intervention for muscle-invasive bladder cancer [1]. Postoperative ileus, that occurs due to various factors such as general anaesthesia, bowel manipulations, and opioid analgesics, is a frequent problem caused by RC which mostly prolongs the length of stay [2]. Extended midline laparotomy incision causes severe pain in patients after surgery and it also affects their physiology [3]. Postoperative pain management is key to reducing the duration of hospitalisation and a patient’s risk of morbidity and mortality [4]. A traditional method of managing postoperative pain is to administer opioid analgesics. However, it causes nausea, constipation, and urinary retention, as well as negative consequences, such as respiratory depression, hormonal and immunological dysfunction, sedation, and postoperative ileus [5]. Transversus abdominis plane (TAP) blockade is a peripheral nerve block of the anterior wall of abdomen. It results in analgesia to the parietal peritoneum and to the skin and muscles of the lower anterior abdominal wall. In TAP block, the afferent neurons of the abdominal wall are blocked by injecting local anaesthesia into the plane between the internal oblique and transverse abdominis muscles, containing the anterior rami of the lower 6 thoracic nerves (T7 to T12) and the 1^st^ lumbar nerve (L1) that innervates the anterior abdominal wall (skin, muscles, and parietal peritoneum) [6]. The advantage of ultrasound guidance is that it enables the direct visualisation of the needle and local anaesthetic placement, thereby improving overall efficacy and safety [7] A single-injection TAP block results in effective analgesia of the abdominal wall but with limited time [8] Despite the fact that catheter techniques outperform systematic opioid use for analgesia, they retain a certain number of complications. For example, they may result in catheter displacement and catheter-relevant infection risk, which could be avoided in selected settings by adding some medications prolonging the blockade duration with single-shot regional anaesthesia techniques [9]. Dexmedetomidine (DD) and fentanyl, as additives to local anaesthesia, have been used in certain cases to achieve superior analgesia and enhance the blockade length in various surgeries [10]. Dexmedetomidine is an alpha-2 agonist that has been approved as a venous sedative and an adjuvant for pain relief  [11]. Dexmedetomidine enhances peripheral nerve block when added to local anesthetics, providing better quality of anesthesia as well as postoperative analgesia [12]. There has been an interest in combining LAs with opioids to improve the duration and quality of peripheral nerve blocks, since the characterization of the opioid receptors in peripheral nerves [13]. Furthermore, various opioids, including fentanyl and sufentanil, have been shown to exert LA-like effects [14]. Because opioid receptors are located on peripheral nerves in the transverse abdominis plane, when LAs are combined with opioids, the duration and quality of this block can be improved [15, 16]. The aim of the current research is to compare dexmedetomidine and fentanyl, as adjuvants to bupivacaine, in the context of US-guided TAP block analgesia among patients who underwent radical cystectomy so as to find the promising candidate for effective postoperative pain management.

## Methods

Following a prospective, randomised and single-centre research design, the current study included a total of 60 patients who underwent RC. The study was conducted at Beni-Suef University Hospital between April 2020 and January 2022. The ethical committee of the Faculty of Medicine, Beni-Suef University (FM-BSU), Egypt, approved the conduct of the study (Identifier: FM-BSU REC/08032020). The current study was registered at Clinical Trials.gov on 23/3/2020 (registration number: NCT04318158). All the participants confirmed their voluntary participation in current study by signing in the written informed consent form. The principles of the Declaration of Helsinki were followed for this study. Sixty patients of ASA grades I and II, from both sexes, aged between 50–70 years of age with a body mass index (BMI) of < 40 kg/m^2^ undergoing RC surgery were included in the study. Patients with ischaemic heart diseases, heart block, congestive heart failure, valvular heart disease, cerebrovascular disease, impaired kidney function, history of chronic liver diseases, coagulation disorders, infection near the site of needle insertion, history of allergic reactions to any of the study medications, previous abdominal surgery, and any neurological or neuromuscular disorder or history of seizures were excluded. The participants were randomly divided into three categories, each containing a total of 20 participants. Group B had patients who received a single shot US-guided TAP block on each side with 20 ml of 0.25% bupivacaine (Sunnypivacaine®, Sunny Pharmaceutical) + 2 ml normal saline; group BF had patients who received a single shot US-guided TAP block on each side with 20 ml of 0.25% bupivacaine + 1 µg/kg fentanyl dissolved in 2 ml normal saline (Fentanyl Hameln®, Sunny Pharmaceutical); and group BD had patients who received a single shot US-guided TAP block on each side with 20 ml of 0.25% bupivacaine + 1 µg/kg dexmedetomidine dissolved in 2 ml normal saline (Precedex®, Hospira Inc). Randomisation was achieved by using computer-generated random numbers, which were then placed in separate opaque envelopes and kept by a data administrator.

### Anaesthetic Technique

All the patients under study underwent routine preoperative check-ups, haematological and biochemical analyses followed by cardiac evaluation. The study protocol was explained to all the participants including visual analogue scale (VAS) on the day of preoperative evaluation. Standard monitoring was established when patients were transferred to the operating room. The patient received midazolam 0.05 mg/kg IV 3 min prior to induction and ondansetron 4 mg IV. Anaesthesia was induced by 2–2.5 mg/kg propofol, 2 μg/kg fentanyl & 0.5 mg/kg atracurium for muscle relaxation. The patient was ventilated using a face mask with 100% oxygen at a rate of 4 L/min and isoflurane 1.2%. After 180 s, the patient was intubated using an appropriately sized cuffed oral tube. Anaesthesia maintenance was performed by isoflurane 1.2% in 100% O_2_ and intravenous fentanyl infusion at a rate of 1–2 μg/kg/hr. Muscle relaxation was continued by atracurium 0.1 mg/kg every 20 min. Mechanical ventilation was performed for all participants to maintain end-tidal carbon dioxide levels between 35–40 mmHg. Intravenous fluid requirements were assessed and provided to patients perioperatively, and normothermia was maintained throughout the surgical procedure. Blood loss was assessed from time to time using visual estimation technique ( blood loss measured in suction canisters and estimated in blood-soaked sponges and drapes) and compensated for.

### Ultrasound-guided TAP procedure

A TAP block was carried out, under US guidance, using the lateral approach after the completion of the surgical procedure and before extubation. The ultrasound used in the study was PHILIPS HD5 whereas the scanning probe was a linear array transducer L12-3 (3–12 MHz). In supine position, the lumbar triangle of Petit (formed anteriorly by external oblique muscle, posteriorly by lattisimus dorsi muscle, and inferiorly by iliac crest) was detected. After complete sterilisation, a linear US probe was transversely placed on the abdominal wall between the costal margin and iliac crest. The findings from above downward indicate the presence of skin, subcutaneous tissue and fat, external oblique, internal oblique, and transversus abdominis muscles. Both peritoneum and bowel loops were visualised deeper in the muscles. Once a TAP was identified between the internal oblique and transversus muscles, a 22-G 80 mm needle (Pajunk SonoPlex® STIM; Geisingen, Germany) was inserted in the plane using USG probe. Upon reaching the plane, 2 ml of saline was injected after negative aspiration of the blood to confirm the correct position of the needle. As categorised earlier, three groups received their respective doses as given herewith; group B had patients who received a single shot US-guided TAP block with 20 ml of 0.25% bupivacaine + 2 ml normal saline; group BF had patients who received a single shot US-guided TAP block with 20 ml of 0.25% bupivacaine + 1 µg/kg fentanyl dissolved in 2 ml normal saline; and group BD had patients who received a single shot US-guided TAP block with 20 ml of 0.25% bupivacaine + 1 µg/kg dexmedetomidine dissolved in 2 ml normal saline. Transverse abdominis plane was visualised by expanding with the injection. Thereafter, same procedure was repeated on the other side.

### Recovery and postoperative management

At the end of the blockade procedure, any residual neuromuscular block was antagonised with neostigmine 0.04 mg/kg and atropine 0.02 mg/kg IV after which extubation was performed. When patients were found to be fully awake and vitally stable, they were transferred to the post-anaesthesia care unit (PACU) and kept under observation for 30 min prior to shifting to the surgical intensive care unit for 24 h.

### Parameter recording

The duration of analgesia after surgery (i.e., time from TAP blockade to the first analgesic request in postoperative period) and the degree of pain (evaluated using VAS) were assessed. An anaesthetist explained the participants on how their pain will be assessed using a VAS scale in the range of 0 (no pain) to 10 (worst pain) [17]. Postoperative VAS score for pain was recorded immediately postoperatively and then at 2, 4, 6, 8, 12, 18, and 24 h at rest and movement. A score ≤ 3 was considered to be acceptable for pain relief. Supplementary rescue analgesia was administered in the form of nalbuphine IV 0.15 mg/kg (at VAS ≥ 4). Total analgesic consumption, in the first 24 h after the blockade, was recorded. The incidence and severity of postoperative complications such as hypotension, bradycardia, respiratory depression, sedation, nausea, and vomiting, during the first 24 h of postoperative period were also recorded. A categorical scoring system (0 = none, 1 = nausea, 2 = retching, and 3 = vomiting) was used to evaluate nausea and vomiting [18]. Sedation scores were evaluated using a sedation scale (0 = awake, 1 = drowsy, 2 = asleep but arousable, 3 = deeply asleep). Patients were considered sedated, if they had a sedation score of > 0 at any time, during the first 24 h after surgery [19]. Patient satisfaction (area of satisfaction was pain relief) was also assessed in the form of 1 = poor, 2 = moderate, 3 = good, and 4 = perfect [20]. *The primary outcome* was the time to the first rescue (TOFS) analgesia after TAP blockade. *The secondary outcomes* were the total dosage of rescue analgesia in the first 24 h after blockade, pain intensity after surgery at rest and movement during the first 24 h using VAS score, and adverse events, if any.

### Sample size calculation

The sample size was assessed by comparing the time of the first analgesic request between patients treated with bupivacaine and dexmedetomidine (BD group), bupivacaine with fentanyl (BF group), and bupivacaine (B group) in ultrasound-guided TAP block in patients, who received RC. According to previous literature [17, 21], the mean ± SD of time to 1^st^ analgesic request among BD participants was 9.8 ± 2.9 h, while in BF participants, it was 5.4 ± 1.5 h and in B group, it was 4.0 ± 0.7 h. So, the minimum sample size was calculated to be 16 patients in each group. In order to find the reduction in VAS score of 0.82 units, 20 samples per group is required with an expected drop rate of 5%. In another study conducted upon dexmedetomidine, the sample size of 20 was used. This sample size is sufficient to determine the real difference of 1 h with 80% power at α = 0.05, using One Way analysis of variance test. G*Power software version 3.1.2 for MS Windows (Franz Faul, Kiel University, Germany) was used to assess the sample size. Therefore, we recruited 20 patients per group to account for dropout.

### Statistical analysis

The findings are described as mean ± standard deviation (± SD), median and range, or frequency (number of cases) and percentage. A Kolmogorov–Smirnov test was used to assess the normality of the numerical data. Group comparisons were carried out using a Kruskal–Wallis test with test as post hoc multiple 2-group comparisons after applying a Bonferroni adjustment for multiple comparisons for numerical data. A chi-square (c^2^) test was used for categorical data. An exact test was used instead when the expected frequency was < 5. Two-sided *p* values under 0.05 were defined as statistically significant. Data analyses were carried out in SPSS version 22.

## Results

**Fig. 1:**
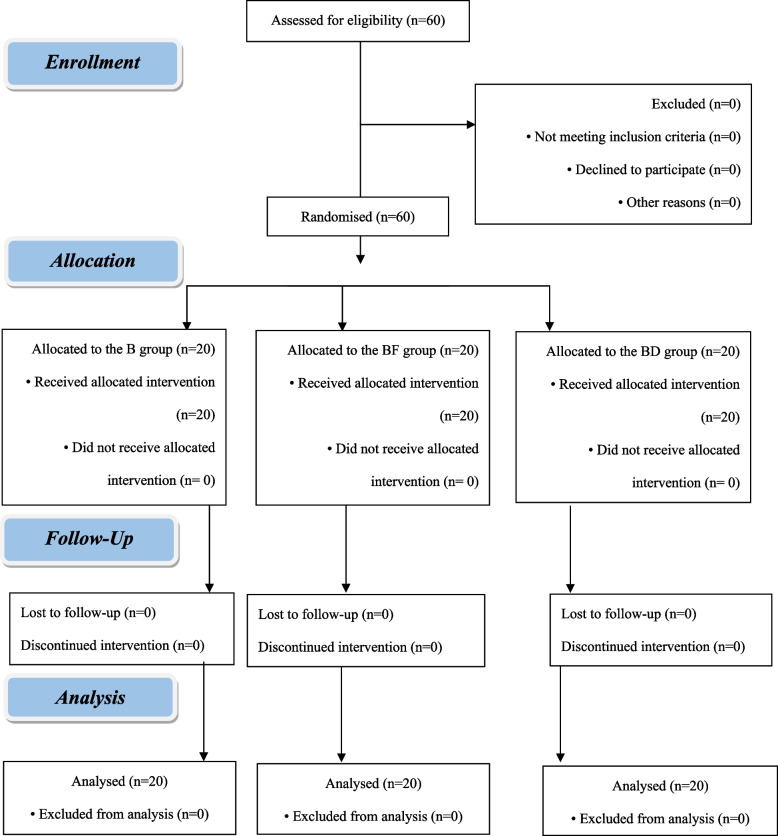
Flow diagram

**Fig. 2:**
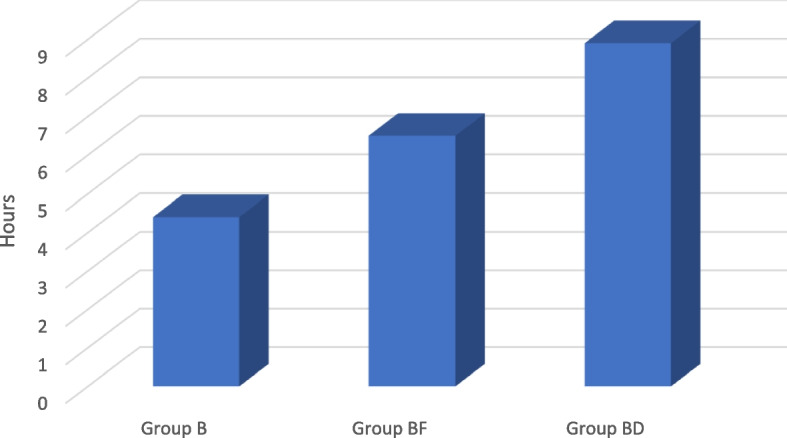
Mean time to 1^st^ rescue analgesia (hours) between the 3 groups

**Fig. 3:**
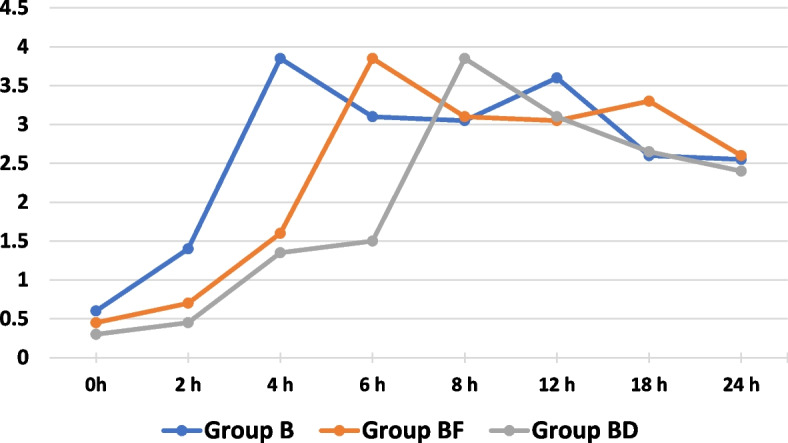
Mean VAS at rest across the different study groups over the study period

**Fig. 4:**
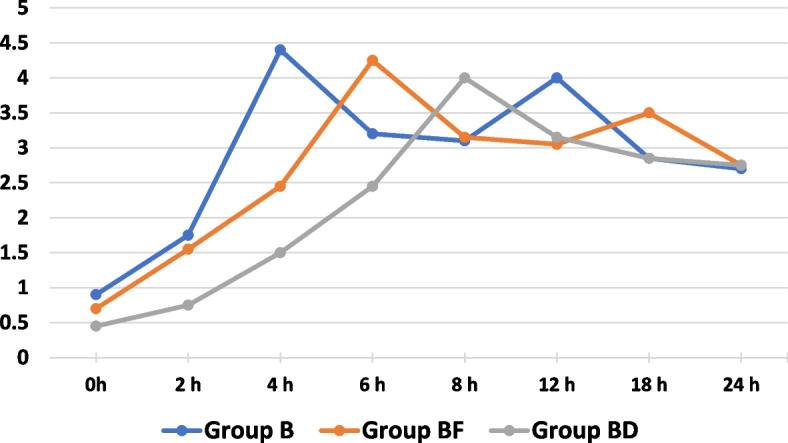
Mean VAS at movement across the different study groups over the study period

In this study, sixty patients were recruited (*n* = 20 per group). All the patients completed the study (Fig. 1). No significant difference was found among the study groups in terms of age, BMI, sex, ASA physical status, and the duration of the operation, as shown in (Table 1). The time of the first request for rescue analgesic was significantly longer in group (BD) (8.90 ± 2.47) than other two groups i.e. (BF) (6.50 ± 1.43) and (B) (4.40 ± 1.05) as shown in (Table 2) and (Fig. 2). Similarly, the total nalbuphine consumption, during the first 24 h, was significantly lower in group (BD) (0.15 ± 0.00) than other two groups i.e. (BF) (0.20 ± 0.07) and (B) (0.24 ± 0.08) which exhibited no significant difference (*P* > 0.05) (Table 2). At different time intervals such as 2, 4, 6, 8, 12, and 18 h postoperatively, the VAS scores at rest had statistically significant difference (*P* < 0.05), whereas no significant difference was found at 0 and 24 h postoperatively (*P* > 0.05). In comparison, no significant difference was found between the groups (B) and (BF) at 2, 4, 6, 12, and 18 h, postoperatively while the groups (B) and group (BD) had statistically significant difference. On the other hand, significant difference was found between (BF) and (BD) at 6, 8, and 12 h postoperatively (Table 3) and (Fig. 3). VAS scores, during movement, secured by the groups at 0, 2, 4, 6, 8, 12, and 18 h postoperatively, had statistically significant difference (*P* < 0.05) whereas it was absent at 24 h postoperatively (*P* > 0.05). However, the groups (B) and (BF) exhibited statistically significant difference at 4, 6, 12, and 18 h postoperatively. On the other hand, the groups (B) and (BD) showcased statistically significant difference at 0, 2, 4, 6, 8, and 12 h postoperatively. The study found no significant difference between the groups (BF) and (BD) at 2, 4, 6, 8, and 18 h postoperatively (Table 4) and (Fig. 4). All the three groups exhibited no significant difference in terms of number of cases with nausea/vomiting (*P* > 0.05) (Table 5). With regards to sedation scores, a significant difference was found among the study groups (*P* < 0.05). However, no significant difference was found between the groups, (B) and (BF) (*P* > 0.05) (Table 5). There was a significant difference found across three groups, in terms of patient satisfaction scores (*P* < 0.05). However, no significant difference was found between the groups, (B) and (BF) (*P* > 0.05) (Table 5).

**Table 1:** Demographic data and operative characteristics among different study groups

	Group B (*n* = 20)	Group BF (*n* = 20)	Group BD (*n* = 20)	*p* value
age (years)	60.40 ± 6.85	59.85 ± 6.59	61.50 ± 6.82	0.704
BMI (kg/m^2^)	24.75 ± 2.31	23.95 ± 2.70	25.30 ± 2.94	0.240
males/females	17/3	18/2	19/1	0.600
ASA I/ II	16/4	15/5	14/6	0.766
operative duration (hours)	6.80 ± 0.77	6.78 ± 0.77	6.40 ± 0.82	0.236

Data are presented as mean ± standard deviation (SD) or as the number of patients

*P*-value < 0.05 (significant)

*P*-value > 0.05 (non-significant)

**Table 2:** Time to first request of rescue analgesic and total nalbuphine consumption/kg during the first post-operative 24 h in the three study groups

	Group B (*n* = 20)	Group BF (*n* = 20)	Group BD (*n* = 20)	*p* value
time to 1st rescue analgesia (hour)	4.40 ± 1.054^**$#**^	6.50 ± 1.43^#^	8.90 ± 2.47	< 0.001
total dose of nalbuphine in 1st 24 h (mg/kg)	0.24 ± 0.08^#^	0.20 ± 0.07^#^	0.15 ± 0.00	< 0.001

Data are represented as mean ± SD (standard deviation)

*P*-value < 0.05 (significant)

*P*-value > 0.05 (non-significant)

^$^ Statistically significant difference compared with group (BF)

^#^ statistically significant difference compared with group (BD)

**Table 3:** Visual analog score changes at rest

	Group B (*n* = 20)	Group BF (*n* = 20)	Group BD (*n* = 20)	*p* value
VAS at rest-0 h	0.60 ± 0.50	0.45 ± 0.51	0.30 ± 0.47	0.167
VAS at rest-2 h	1.40 ± 0.50^**$#**^	0.70 ± 0.47	0.45 ± 0.51	< 0.001
VAS at rest-4 h	3.85 ± 0.37^**$#**^	1.60 ± 0.50	1.35 ± 0.49	< 0.001
VAS at rest-6 h	3.10 ± 0.31^**$#**^	3.85 ± 0.37^#^	1.50 ± 0.51	< 0.001
VAS at rest-8 h	3.05 ± 0.22^#^	3.10 ± 0.31^#^	3.85 ± 0.37	< 0.001
VAS at rest-12 h	3.60 ± 0.50^**$#**^	3.05 ± 0.22	3.10 ± 0.31	< 0.001
VAS at rest-18 h	2.60 ± 0.50^**$**^	3.30 ± 0.47^#^	2.65 ± 0.59	< 0.001
VAS at rest-24 h	2.55 ± 0.51	2.60 ± 0.50	2.40 ± 0.50	0.426

Data are represented as mean ± SD (standard deviation)

*P*-value < 0.05 (significant)

*P*-value > 0.05 (non-significant)

^$^ Statistically significant difference compared with group (BF)

^#^ statistically significant difference compared with group (BD)

**Table 4:** Visual analog score changes at movement

	Group B (*n* = 20)	Group BF (*n* = 20)	Group BD (*n* = 20)	*p* value
VAS at movement-0 h	0.90 ± 0.31^#^	0.70 ± 0.47	0.45 ± 0.51	0.010
VAS at movement-2 h	1.75 ± 0.44^#^	1.55 ± 0.51^#^	0.75 ± 0.44	< 0.001
VAS at movement-4 h	4.40 ± 0.75^**$#**^	2.45 ± 0.51^#^	1.50 ± 0.51	< 0.001
VAS at movement-6 h	3.20 ± 0.62^**$#**^	4.25 ± 0.72^#^	2.45 ± 0.51	< 0.001
VAS at movement-8 h	3.10 ± 0.45^#^	3.15 ± 0.49^#^	4.00 ± 0.56	< 0.001
VAS at movement-12 h	4.00 ± 0.92^**$#**^	3.05 ± 0.22	3.15 ± 0.49	< 0.001
VAS at movement-18 h	2.85 ± 0.37^**$**^	3.50 ± 0.83^#^	2.85 ± 0.49	0.003
VAS at movement-24 h	2.70 ± 0.47	2.75 ± 0.44	2.75 ± 0.44	0.920

Data are represented as mean ± SD (standard deviation)

*P*-value < 0.05 (significant)

*P*-value > 0.05 (non-significant)

^$^ Statistically significant difference compared with group (BF)

^#^ statistically significant difference compared with group (BD)

**Table 5:** Postoperative nausea/vomiting score, sedation score and patient satisfaction score in the three study groups

	Group B (*n* = 20)	Group BF (*n* = 20)	Group BD (*n* = 20)	*p* value
Nausea and vomiting:				
No nausea/vomiting	15 (75.0%)	14 (70.0%)	17 (85.0%)	0.69
Nausea	2 (10.0%)	2 (10.0%)	2 (10.0%)	
Retching	1 (5.0%)	3 (15.0%)	1 (5.0%)	
Vomiting	2 (10.0%)	1 (5.0%)	0 (0.0%)	
Sedation:				
Awake	9 (45.0%)	13 (65.0%)	19 (95.0%)	0.015
Drowsy	8 (40.0%)	6 (30.0%)	1 (5.0%)	
Asleep but arousable	3 (15.0%)	1 (5.0%)	0 (0.0%)	
Patient satisfaction:				
Poor satisfaction	4 (20.0%)	2 (10.0%)	0 (0.0%)	< 0.001
Moderate	10 (50.0%)	6 (30.0%)	1 (5.0%)	
Good	5 (25.0%)	9 (45.0%)	5 (25.0%)	
Perfect	1 (5.0%)	3 (15.0%)	14 (70.0%)	

Data are represented as numbers and percent

*P*-value < 0.05 (significant)

*P*-value > 0.05 (non-significant)

## Discussion

Many reports have demonstrated effective analgesia, using TAP block, for postoperative pain management. Using ultrasound guidance, TAP block can be carried out with minimal risk. Several adjuvants are used to enhance the duration and intensify the quality of local anaesthesia using different regional block techniques and peripheral nerve blocks. The current randomised study was conducted to compare the outcomes of DD and fentanyl, as adjuvants to bupivacaine, in US-guided TAP block analgesia among patients who underwent RC, for postoperative analgesic efficacy. In current study, the time taken for first postoperative rescue analgesia was longer in DD/bupivacaine group than the fentanyl/bupivacaine and bupivacaine alone groups. DD/bupivacaine group had a significantly low postoperative VAS score than the other two groups, over the course of first 24 h postoperatively. The incidence of complications, related to nalbuphine consumption (sedation), was significantly lower in DD/bupivacaine TAP block group compared to other two groups. Further, DD/bupivacaine TAP block group also experienced a significant improvement in patient satisfaction. The three groups did not differ significantly in terms of the number of cases with nausea/vomiting. The review of literature failed to find any research study conducted upon safety and efficacy of DD versus fentanyl, as adjuvants to bupivacaine, in TAP blocks for patients who underwent RC. Nevertheless, its efficacy in TAP block was already probed in the context of several surgical procedures such as, laparoscopic hernia repair, hysterectomy, and caesarean section. Many studies have reported that the addition of DD to local anaesthetics can extend the duration of postoperative analgesia than the local anaesthetics alone. Coinciding with our results, In the study conducted by *R. Aksu and colleagues* [22], the efficacy of bupivacaine and its association with DD in TAP block US-guided surgery were assessed among patients who underwent abdominal surgery. The study found that the addition of DD to bupivacaine in TAP block reduced postoperative analgesic requests and less VAS scores at all time points during 24 h postoperative period. The group which had DD with bupivacaine secured high postoperative patient satisfaction scores compared to the rest of the groups. It also demonstrated that DD does not influence nausea and vomiting scores and antiemetic requirements. In a randomised controlled study, *Neethirajan *et al*.* [23] demonstrated that DD, as an adjuvant to bupivacaine in TAP block among patients who underwent laparoscopic appendicectomy, provided prolonged postoperative analgesia against the usage of bupivacaine alone. Patients with DD secured significantly low pain scores. In a recent systematic review and meta-analysis that was conducted focusing 20 published trials and 1,212 cases that met the inclusion criteria, *Sun and colleagues *[24] demonstrated that the addition of DD, to any LA agent in TAP block, significantly reduced the pain score 8 h after surgery at rest, 4 h after surgery on movement, and opioid consumption in comparison with the control group, who received the LA agent alone. The authors found no significant effects on the incidence of nausea and vomiting, hypotension, bradycardia, somnolence, or pruritus after surgery. In other terms, the study found that DD, as an adjuvant, resulted in better postoperative analgesia. Further, it also reduced postoperative analgesics and prolonged the effect of LA, when administered in TAP blocks for abdominal surgery. Supporting our study, *Xue *et al*.* [11], in their study, categorised 90 patients under three groups as described here with; group I received postoperative intravenous analgesia only after general anaesthesia; group II received TAP block with 20 ml of 0.375% ropivacaine; and group III received TAP block with 20 ml of 0.375% ropivacaine and 1 μg/kg DD after induction. The authors identified that US-guided TAP block, in combination with DD as an adjuvant, improved the recovery from anaesthesia as the time of awakening, spontaneous breathing, and extubation were found to be significantly reduced. Further, it also reduced the postoperative pain during gynaecological laparoscopy. In alignment with these results, DD was investigated by *Bansal and Sood* [25] among 40 patients who underwent caesarean section (C-section). The aim of the study was to investigate the effect of adding DD to ropivacaine, in US-guided TAP block, among caesarean delivery patients postoperatively. The study established that DD, when added to ropivacaine in TAP block, prolonged the ToFS analgesia. *Varshney *et al*.* [26] compared the effects of adding DD to levobupivacaine in TAP block with US-guided technique for caesarean delivery. The authors found that the addition of DD increased the duration of analgesia and improved the patient outcomes in comparison with TAP block with levobupivacaine alone. Partially consistent with our study, *Abdelraouf and colleagues* [27] demonstrated that the addition of DD to bupivacaine, for TAP block patients, has been listed for elective caesarean surgery with subarachnoid blockade delayed TOFS analgesia. On the other hand, the study found that the addition of fentanyl to bupivacaine resulted in no added advantage. In another study, *WANG *et al*. *[28] checked whether the addition of fentanyl, to TAP block procedure, enhances the analgesic duration after C-section. The authors revealed that there was no improvement in TAP block analgesia, after caesarean, section under spinal anaesthesia. Furthermore, in a study conducted upon patients who underwent elective lower abdominal surgeries, *Metwally and colleagues* [21] found that the addition of fentanyl to local anaesthesia in ultrasound-guided TAP block, prolonged the time for first rescue. Many studies such as *Mane and colleagues *[29], *Chavan and colleagues *[30], and *Sert and colleagues *[31] have inferred that the addition of fentanyl to local anaesthetics improved the quality and duration of peripheral nerve blocks. However, some other studies such as *Kalso *et al*.* [32] and *Magistris *et al*.* [33] demonstrated that the addition of fentanyl or other opioids to local anaesthetics brought no significant and clinically relevant advantage for peripheral nerve blocks. Conflicting our study, *Joseph *et al*.* [34] compared the effects of fentanyl and DD, as adjuvants to ropivacaine, in US‑guided TAP block for pain management after caesarean section under spinal anaesthesia. The authors found no considerable difference, across the study groups, in terms of duration of analgesia produced by addition of DD or fentanyl to ropivacaine. Against our study, *Ding W and colleagues *[35] investigated the patients who were scheduled for gastrectomy and found that the addition of DD to local anaesthetic showed no significant extension in the duration or enhancement in the quality of analgesia of TAP block. In most studies performed with addition of dexmedetomidine to local anesthetic, patients have been subjected to general anesthesia, and the TAP block performed after general anesthesia. So, The drowsiness of patients after waking up have also been attributed to general anesthesia. Ramya et al. [36] used spinal anesthesia and did not indicate that patients fell asleep. The use of 1 and 1.5 µ/kg dexmedetomidine in the TAP block provides a longer analgesic effect and reduces the need for postoperative analgesics in comparison with its use at 0.5 µ/kg. However, dexmedetomidine at 1.5 µ/kg causes more adverse effects, like drowsiness, bradycardia, and lower MAP than dexmedetomidine at 1 µ/kg. According to the results of this study, the appropriate dose of dexmedetomidine for adding bupivacaine in the TAP block is 1µ/kg [37]. Till date, no studies on human subjects have shown the presence of neurotoxicity when dexmedetomidine was used [38]. It should be noted that many trials examining peripheral administration of opioids reported side effects typical of systemic administration, including pruritus, nausea,and vomiting [39]. Opioids are known to produce nausea, vomiting, sedation, respiratory depression and itching. However, Manju Lata Shakya et al. [40] found that the incidence of all these side effects wasn’t present. It may be inferred that fentanyl had relatively minor systemic absorption when used as an adjuvant to local anaesthetic in TAP block. Our study has a certain number of limitations. First, the sample size was relatively small, and for greater accuracy, studies need to be conducted with a larger sample size. Second, we were unable to measure the onset time of TAP block because the patients did not fully recover from general anesthesia. Third, we were not able to assess serum DD and fentanyl concentrations to probe whether the analgesia can be attributed to systemic absorption or potentiation of the LA effect. For this reason, we couldn’t assess the systemic effects of DD. Finally, further studies are warranted to best assess the efficacy of TAP versus other regional techniques for postoperative analgesia and satisfaction in patients undergoing different types of surgeries.

## Conclusions

DD as an adjuvant to bupivacaine, compared with fentanyl, was associated with prolonged postoperative analgesia, as well as a lower requirement of postoperative analgesics over the course of the first 24 h. In addition, it increases satisfaction in patients undergoing RC. Moreover, it did not result in marked sedation or adverse effects.

## Data Availability

The datasets used and analysed during the current study are available from the corresponding author upon reasonable request.
